# Online socializing among men who have sex with men and transgender people in Nairobi and Johannesburg and implications for public health‐related research and health promotion: an analysis of qualitative and respondent‐driven sampling survey data

**DOI:** 10.1002/jia2.25603

**Published:** 2020-10-01

**Authors:** Elizabeth Fearon, Adam Bourne, Siyanda Tenza, Thesla Palanee‐Phillips, Rhoda Kabuti, Peter Weatherburn, Will Nutland, Joshua Kimani, Adrian D Smith

**Affiliations:** ^1^ Department of Global Health & Development London School of Hygiene & Tropical Medicine London United Kingdom; ^2^ Australian Research Centre in Sex, Health & Society La Trobe University Melbourne Australia; ^3^ Wits Reproductive Health and HIV Institute School of Clinical Medicine University of the Witwatersrand Johannesburg South Africa; ^4^ Partners for Health and Development Nairobi Kenya; ^5^ Sigma Research Department of Public Health, Environments and Society London School of Hygiene & Tropical Medicine London United Kingdom; ^6^ Department of Community Health Sciences University of Manitoba (UoM) Winnipeg Canada; ^7^ Nuffield Department of Population Health University of Oxford Oxford United Kingdom

**Keywords:** men who have sex with men, transgender persons, social media, internet, HIV, sexual health

## Abstract

**Introduction:**

There is little published literature about gay, bisexual and other men who have sex with men and transgender individuals (MSM and TG)’s use of social media in sub‐Saharan Africa, despite repressive social and/or criminalizing contexts that limit access to physical HIV prevention. We sought to describe MSM and TG’s online socializing in Nairobi and Johannesburg, identifying the characteristics of those socializing online and those not, in order to inform the development of research and health promotion in online environments.

**Methods:**

Respondent‐driven sampling surveys were conducted in 2017 in Nairobi (n = 618) and Johannesburg (n = 301) with those reporting current male gender identity or male sex assigned at birth and sex with a man in the last 12 months. Online socializing patterns, sociodemographic, sexual behaviour and HIV‐testing data were collected. We examined associations between social media use and sociodemographic characteristics and sexual behaviours among all, and only those HIV‐uninfected, using logistic regression. Analyses were RDS‐II weighted. Thirty qualitative interviews were conducted with MSM and TG in each city, which examined the broader context of and motivations for social media use.

**Results:**

Most MSM and TG had used social media to socialize with MSM in the last month (60% Johannesburg, 71% Nairobi), mostly using generic platforms (e.g. Facebook), but also gay‐specific (e.g. Grindr). HIV‐uninfected MSM and TG reporting riskier recent sexual behaviours had raised odds of social media use in Nairobi, including receptive anal intercourse (adjusted OR = 2.15, *p* = 0.006), buying (aOR = 2.24, *p* = 0.015) and selling sex with men (aOR = 2.17, *p* = 0.004). Evidence for these associations was weaker in Johannesburg, though socializing online was associated with condomless anal intercourse (aOR = 3.67, *p* = 0.003) and active syphilis (aOR = 13.50, *p* = 0.016). Qualitative findings indicated that while online socializing can limit risk of harm inherent in face‐to‐face interactions, novel challenges were introduced, including context collapse and a fear of blackmail.

**Conclusions:**

Most MSM and TG in these cities socialize online regularly. Users reported HIV acquisition risk behaviours, yet this space is not fully utilized for sexual health promotion and research engagement. Effective, safe and acceptable means of using online channels to engage with MSM/TG that account for MSM and TG’s strategies and concerns for managing online security should now be explored, as complements or alternatives to existing outreach.

## 1 INTRODUCTION

Access to the Internet, smartphones, online platforms and social media is rising rapidly in sub‐Saharan Africa (SSA), particularly among young people, men and those with a higher education [1, 2]. Gay, bisexual and other men who have sex with men and transgender people (MSM and TG) have been disproportionately affected by HIV around the world, including in generalized epidemic settings in East and Southern Africa [3, 4, 5, 6]. The use of social media for socializing and partner‐seeking among gay men and other MSM is well‐documented and described in many parts of the world [7, 8]. Behavioural interventions delivered on Internet and social media platforms have demonstrated impact on sexual health among MSM in the United States, East Asia and Peru [9] and are adaptable to changing technology and HIV prevention tools [10]. However, despite the severity of HIV epidemics among MSM and TG in parts of SSA [5] and the high prevalence of stigma and discrimination towards MSM and TG in many African countries [4, 25], sexual health interventions rarely harness this route of HIV prevention delivery and there are few validated online interventions [11, 12].

There is evidence that MSM in SSA do use social media [13] and many do so to seek partners. Surveys of MSM from urban Lesotho (2011) and eSwatini (2014) found that 39% and 44% of MSM, respectively, reported having met a sexual partner online [14], and in Nigeria (2013 to 2015), 62% reported having found sexual partners online [15]. Online partner‐seeking varied by educational attainment, religion, age, sexual and gender identity and size of social network of MSM.

To further consider the utility of Internet or app‐based methods to complement existing approaches to surveillance, intervention delivery and research engagement among MSM and TG in SSA, we need better understanding of online use within broader social and sexual networking. The Internet has been successfully used in other regions to survey MSM [16, 17], and proved to be a cost‐effective method to engage large and broadly representative population samples [18]. In SSA, there are examples of surveys conducted entirely online [19, 20] or that utilize a combination of online surveys and referral from community organizations [21]. However, the representativeness of MSM and TG who socialize online to wider MSM and TG populations in SSA remains unclear, and prompts concern that employing online environments for research sampling or intervention delivery may only reach a relatively affluent and educated sub‐population [22].

Online engagement with MSM and TG populations must also navigate the dual role that social media can play in safety and security [23]. Studies have highlighted how online socializing can be viewed as safer than meeting in physical locations [14, 15, 24]. This is unsurprising in a region where same‐gender sexual relationships are usually criminalized, and where not, remain highly stigmatized [25, 26, 27, 28, 29]. Physical venues used for socialization in the region are often covert and short‐lived as they are targeted by police, authorities or unofficial groups [30]. However, while providing physical security, online socializing and partner‐seeking may present other risks that MSM and TG must negotiate, including blackmail from partners met online [24]. How MSM and TG in SSA weigh up and mitigate the risks and benefits of in‐person versus online socializing and partner‐seeking is currently understudied.

Here we have sought to: (1) examine the extent, nature and means of engagement among MSM and TG individuals who use social media in Johannesburg, South Africa and Nairobi, Kenya; (2) identify their demographic and sexual behaviour‐related characteristics and (3) identify opportunities to use social media settings to facilitate both public health research and health promotion interventions.

## 2 METHODS

### 2.1 Population and settings

We used mixed methods to describe and investigate qualitative and population‐representative quantitative survey data collected from MSM and transgender individuals from Nairobi and Johannesburg from 2016 to 2017. MSM and TG were eligible if they were aged at least 18 years; reported consensual sex with a man in the previous 12 months; resided in Johannesburg or within 50 km of Nairobi county; and either currently identified as a man or had been assigned male sex at birth.

### 2.2 Data Collection

#### 2.2.1 Phase 1 qualitative

We recruited 30 MSM/TG at each site (total n = 60) between May 2016 and July 2017 for an in‐depth face‐to‐face interview. They were purposively selected to provide diversity in age, socio‐economic status and ethnicity. Participants were recruited through existing community outreach activities, community organizations and sexual health clinics. Interviews lasting 60 to 90 minutes were conducted in English, Kiswahili (Kenya) or Zulu (South Africa), depending on the preference of the participant, and were audio‐recorded, transcribed and translated prior to data coding. In addition to issues relating to sexual health and HIV, the interviews explored how men met and engaged with other men in both physical and online environments.

#### 2.2.2 Phase 2 respondent driven sampling surveys

Respondent‐driven sampling (RDS) surveys [31] were conducted in Nairobi (April ‐December 2017) and Johannesburg (April ‐ November 2017) with intended sample sizes of 600 and 300 respectively. There were 10 initial “seed” participants in Nairobi and nine in Johannesburg (not all productive). Participants were given two coupons for onwards recruitment valid for two weeks. They were reimbursed for their participation and for each enrolled recruit. More detail about RDS recruitment in Johannesburg is given elsewhere [32].

After screening, participants gave informed consent, privately completed a self‐administered questionnaire on a tablet computer, undertook HIV counselling and testing according to national guidelines, and visited a clinician for examination, blood draw and where indicated STI treatment, linkage to HIV treatment services or for pre‐exposure prophylaxis. Blood samples were tested for active syphilis (Treponema pallidum haemmaglutination (TPHA) & Rapid Plasma Reagin (RPR) and HIV viral load (GeneXpert HIV‐1 VL) if HIV‐seropositive.

The questionnaire included sociodemographics, sexual identity and gender identity, online and in‐person socializing patterns and social network size (number of adult MSM in the relevant city with whom the participant had spoken in the previous two weeks). For the previous three months, participants reported whether they had sex with a man and/or a woman; what types of sex they had had; and frequencies of condom use during anal and vaginal intercourse. For the previous 12 months they reported: the number of sexual partners they had had; whether they had received money, gifts or favours in exchange for sex with men and with women; and whether they had given gifts, money or favours in exchange for sex with men. Participants reported whether they had experienced urethral or rectal STI symptoms over that period.

Participants self‐reported if they had socialized online with other MSM in the previous month; previous year; more than one year ago, or never. If any use was reported, they were asked to indicate all the sites they had visited or apps that they had used in the previous month from a list populated with all sites/apps known to operate in the country, with space to add options. Social media were further categorized as generic (sites/apps in widespread use among the whole population, e.g. Facebook, Instagram), gay‐specific (sites/apps targeted to MSM only, e.g. Grindr, Planet Romeo) and dating‐specific (sites/apps targeted to users seeking sex or relationships, e.g. Badoo).

### 2.3 Analysis

#### 2.3.1 Qualitative analysis

Digital recordings of the interviews were transcribed verbatim and translated to English. Debriefing reports were written and discussed after the interview to identify emergent themes. Interview transcripts were subject to a detailed thematic analysis [33], supported by NVIVO 10. Transcripts were read and re‐read by a panel of researchers and interviewers to identify initial codes (relevant or significant features) that comprised the coding framework. The meaning and conceptual distinction of these codes was discussed and agreed upon among the qualitative research team, following which all sections of each transcript were coded using this framework. Data within each code were then carefully reviewed and formulated into higher level themes and cross‐referenced against the rest of the coding framework for conceptual clarity.

#### 2.3.2 Quantitative analysis

We report findings from each city separately. All percentages are weighted using RDS‐II estimation [34] using the self‐reported measure of social network degree. We examined sociodemographic associations with online socializing within the previous month using logistic regression, dropping seed participants and probability weighting by inverse network size, and used Wald tests to assess statistical evidence for associations. We then examined the associations between online socializing and sexual behaviours, first among all MSM and TG in each city, then restricted to those HIV‐uninfected to assess the association between online socializing and measures of behavioural HIV acquisition risk. We first examined crude associations, then adjusted for sociodemographic characteristics found to be associated with online socializing and age (Model 1), and finally examined associations with sexual behaviours, partners and STIs among only HIV‐uninfected participants (Model 2). For multivariate models, covariates with *p* < 0.100 in bivariate associations were retained.

### 2.4 Ethics

Ethical approval was obtained from the London School of Hygiene and Tropical Medicine for both sites, the Human Research Ethics Committee of the University of the Witwatersrand for Johannesburg and the Kenya Medical Research Institute and University of Oxford for Nairobi.

## 3 RESULTS

### 3.1 Quantitative findings

#### 3.1.1 Characteristics of MSM and TG

A total of 618 MSM and TG participants were recruited in Nairobi and 301 MSM and TG participants in Johannesburg. Convergence of reporting social media engagement was achieved (Figure S1).

The majority of MSM and TG in Nairobi and Johannesburg were young and self‐identified as gay/homosexual and cisgender (Table 1). Most had completed secondary education, yet many were unemployed. Most MSM and TG had been sexually active with another man in the previous three months. Nearly two‐fifths of men in Nairobi and one‐fifth of those in Johannesburg had sold sex to a man within the previous 12 months.

**Table 1:** Characteristics of MSM and TG in Nairobi and Johannesburg

Characteristics	Nairobi			Johannesburg		
	n	Unweighted %	RDS %	n	Unweighted %	RDS %
Age group in years						
18 to 21	162	26.2	28.3	75	24.9	24.1
22 to 24	177	28.6	28.6	67	22.3	23.8
25 to 29	136	22.0	20.9	72	23.9	22.7
30+	143	23.1	22.2	87	28.9	29.5
Born in Nairobi/Johannesburg						
Born in Nairobi/Johannesburg	179	29.0	30.8	182	60.5	62.0
Born elsewhere in Kenya/South Africa	299	48.4	47.7	98	32.6	31.6
Born outside Kenya/South Africa	123	19.9	21.5	18	6.0	6.5
Religion						
Christianity	536	86.7	89.5	260	86.4	83.7
Islam	53	8.6	7.6	4	1.3	1.5
Other	4	0.6	0.3	1	0.3	0.0
None	18	2.9	2.6	35	11.6	14.8
Neighbourhood (Nairobi)						
Dagoretti	95	15.4	16.0			
Embakasi	146	23.6	23.7			
Kamukunji	18	2.9	2.9			
Kasarani	111	18.0	18.7			
Langata	34	5.5	5.6			
Makadara	18	2.9	2.2			
Starehe	83	13.4	13.3			
Westlands	53	8.6	8.2			
Outskirts	53	8.6	8.1			
Missing	7	1.1	1.2			
Neighbourhood (Johannesburg)						
Soweto				158	52.5	55.3
Hillbrow				44	14.6	16.5
Brammfontein				28	9.3	7.4
Orange Farm				13	4.3	4.0
Other^a^				58	19.3	16.7
Sexual identity						
Gay	448	72.5	73.2	216	71.8	70.2
Bisexual	143	23.1	23.4	71	23.6	26.5
Heterosexual	2	0.3	0.4	3	1.0	0.8
Other, None, Don't know	16	2.6	2.9	9	3.0	2.5
Gender Identity^b^						
Cisgender male	528	85.4	86.2	233	77.4	78.3
Transfeminine	70	11.3	11.3	45	15.0	13.2
Transmasculine	3	0.5	0.4	2	0.7	0.3
Non‐binary	17	2.8	2.1	21	7.0	8.2
Monthly income (Nairobi)						
<5000 KSH	224	36.2	37.8			
5000 to 9999 KSH	166	26.9	25.6			
10,000 to 19,999 KSH	129	20.9	20.5			
20,000 KSH +	55	8.9	8.6			
Monthly income (Johannesburg)						
0 to 499 ZAR				82	27.2	28.1
500 to 999 ZAR				39	13.0	17.5
1000 to 1999 ZAR				57	18.9	19.4
2000 to 4999 ZAR				74	24.6	24.4
5000 + ZAR				30	10.0	10.5
Employment status						
Employed full‐time	57	9.2	8.8	32	10.6	8.8
Employed part‐time	122	19.7	19.2	42	14.0	15.0
Self‐employed	159	25.7	27.4	33	11.0	9.7
Unemployed	247	40.0	41.7	168	55.8	59.3
Student	12	1.9	1.6	19	6.3	5.3
Other	11	1.8	1.3	6	2.0	1.9
Missing	44	7.1	7.6			
Completed educational attainment						
Primary	111	18.0	18.1	25	8.3	8.2
Secondary	329	53.2	55.0	200	66.4	68.8
Higher education	171	27.7	26.9	75	24.9	23.1
Marital status						
Not married	496	80.3	81.8	267	88.7	88.3
Married to a man or transgender individual	65	10.5	10.8	30	10.0	11.1
Married to a woman	50	8.1	7.4	2	0.7	0.6
*Online socializing with MSM*						
Last time socialized online with MSM using social media, website or mobile app.						
In the last month	461	74.6	70.9	201	66.8	60.1
In the last year but not the last month	40	6.5	7.6	31	10.3	11.2
More than one year ago	43	7.0	7.2	21	7.0	7.2
Never	74	12.0	14.3	48	15.9	21.5
*Sexual behaviours*						
Sex with a man (three months)	543	87.9	87.2	234	77.7	75.7
Sex with a woman (three months)	174	28.2	28.3	82	27.2	31.7
Condomless anal intercourse (3 months)	265	42.9	41.8	113	37.5	35.9
Receptive anal sex (three months)	321	51.9	49.0	143	47.5	43.0
Number of sexual partners (three months)						
0	75	12.1	12.8	67	22.3	24.3
1	148	23.9	28.3	94	31.2	35.8
2	171	27.7	30.6	70	23.3	22.2
3 to 5	154	24.9	20.9	52	17.3	13.9
6+	70	11.3	7.3	18	6.0	3.9
Sold sex to a man (12 months)	297	48.1	43.8	69	22.9	21.9
Bought sex from a man (12 months)	177	28.6	28.2	31	10.3	10.0
STI symptoms (12 months)	225	36.4	35.2	301	100.0	28.6
Syphilis (active, RPR and TPHA positive)	5	0.8	1.1	28	9.3	9.7
CT (urethral)	39	6.3	7.3	18	6.0	6.2
NG (urethral)	27	4.4	4.4	4	1.3	1.6
CT (rectal)	53	8.6	8.1	–	–	–
NG (rectal)	76	12.3	13.2	–	–	–

RDS‐II weighted percentage given (inverse network size weighting, seed participants dropped). Seed participants are included in frequency counts. n’s do not add to full sample size where responses were missing. CT, Chlamydia trachomatis; KSH, Kenyan shillings (currency); MSM/ and TG, men who have sex with men and transgender people; NG, Neisseria gonorrhoeae; RPR and TPHA positive, Rapid Plasma Reagin and Treponema pallidum haemmaglutination positive, indicating active syphilis infection; ZAR, South African Rand (currency).

^a^ Other Johannesburg neighbourhoods include all those with fewer than 10 participants each

^b^ Gender identity was assessed using what at the time was considered best practice via a two‐step approach [35], comprising assessment of sex assignment at birth (male, female or prefer not to say) and current gender identity (male, female, transgender or none of these). Alongside recommendations [36], we described participants as transmasculine where they had been assigned female sex at birth, but now identified as male or transgender, and transfeminine where they had been assigned male sex at birth, but now identified as female or transgender. Participants who did not currently identify as male, female or transgender were described as ‘Non‐binary’.

#### 3.1.2 Use of social media apps and sites for socializing with other MSM

Most participants reported having socialized online with MSM in the previous month, significantly higher in Nairobi than Johannesburg (70.9%; 95% CI 66.5 to 75.0 and 60.1%; 95% CI 53.2 to 66.6 respectively; *p < *0.0068). Few reported never having done so: 14.3% in Nairobi and 21.5% in Johannesburg.

#### 3.1.3 Sites/apps used for socializing with MSM

**Figure 1:**
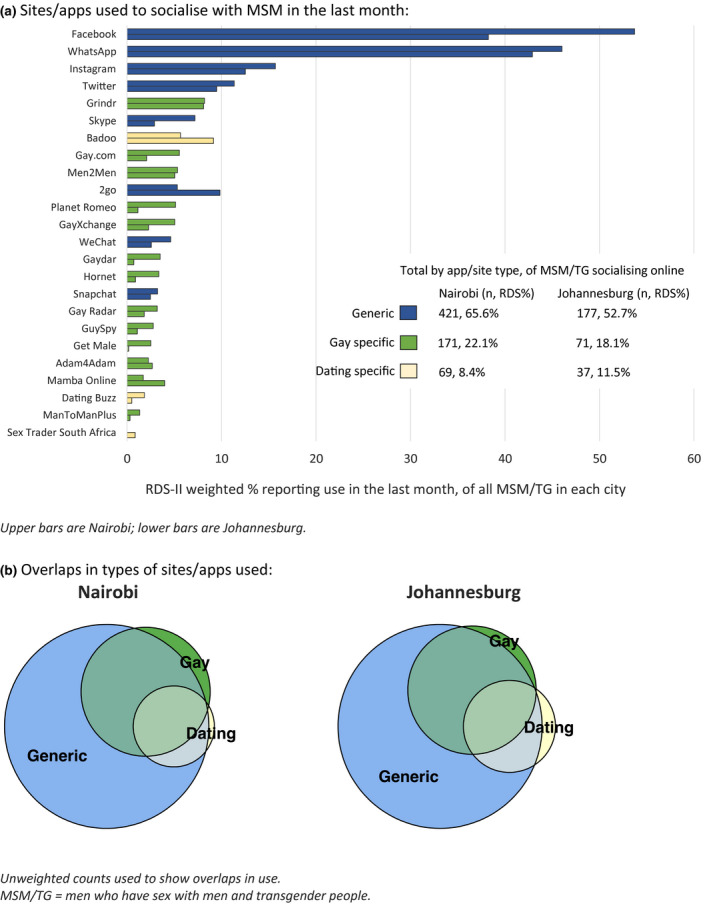
Sites/apps used to socialize with MSM among MSM and TG in Nairobi (n = 618) and Johannesburg (n = 301) during the month prior to interview. **(A)** Sites/apps used to socialize with MSM in the last month. Upper bars are Nairobi; lower bars are Johannesburg. **(B)** Overlaps in types of sites/apps used. Unweighted counts used to show overlaps in use. MSM/TG, men who have sex with men and transgender people.

The most popular sites/apps used for socializing with MSM in the last month were similar in both cities. Generic social media sites/apps were the most widely used, notably Facebook (53.7% Nairobi; 38.2% Johannesburg) and WhatsApp (46.0% and 42.9%, respectively, Figure 1). Fewer participants used gay‐ or dating‐specific apps, nearly all of whom also used generic apps/sites to socialize with MSM (Figure 1B). Grindr was the most frequently cited gay‐specific service in both cities, used by 8.2% (Nairobi) and 8.1% (Johannesburg) in the previous month.

#### 3.1.4 Characteristics associated with online socializing with MSM in the previous month

Few sociodemographic differences were associated with recent online socializing with MSM, and these were inconsistent across cities (Table 2).

**Table 2:** Associations between socializing online with MSM in the previous month and sociodemographic characteristics of MSM and TG in Nairobi and Johannesburg

Characteristic	Nairobi, n = 608 without seeds											Johannesburg, n = 292, without seeds									
			Crude					Adjusted						Crude				Adjusted			
	n	RDS %	OR		95% CI		*p* value	aOR	95% CI		*p* value	n	RDS %	OR	95% CI		*p* value	aOR	95% CI		*p* value
Overall socialized online with other MSM in the last month	461/618	70.9										201/301	60.1								
Age group in years																					
18 to 21	112/162	64.0	1.00				0.133	1.00			0.114	55/75	67.0	1.00			0.319	1.00			0.236
22 to 24	134/177	73.2	1.54		0.89	2.65		1.55	0.89	2.73		47/67	58.9	0.71	0.31	1.63		0.66	0.28	1.53	
25 to 29	111/136	78.5	2.05		1.10	3.84		2.23	1.15	4.34		48/72	65.6	0.94	0.41	2.17		0.94	0.39	2.24	
30+	104/143	69.5	1.28		0.73	2.26		1.49	0.82	2.71		51/87	51.2	0.52	0.24	1.13		0.46	0.20	1.08	
Born in Nairobi/Johannesburg																					
Born in Nairobi/Johannesburg	124/179	66.6	1.00				0.44					113/182	54.8	1.00			0.052	1.00			0.073
Born elsewhere in Kenya/South Africa	226/299	71.9	1.28		0.80	2.06						75/98	73.2	2.25	1.16	4.36		2.29	1.10	4.78	
Born outside Kenya/South Africa	98/123	74.0	1.43		0.78	2.61						12/18	53.9	0.87	0.29	3.18		1.12	0.30	4.17	
Religion																					
Christianity	404/536	72.0	1.00				0.002	1.00			0.004	175/260	60.9	1.00			0.192				
Islam	42/53	75.8	1.22		0.54	2.75		1.25	0.58	2.72		2/4	17.8	0.14	0.02	1.17					
Other	1/4	8.5	0.04		0.00	0.54		0.04	0.00	0.49		0/1	0.0								
None	10/18	32.7	0.19		0.06	0.56		0.20	0.06	0.65		23/35	59.0	0.92	0.40	2.13					
Neighbourhood (Nairobi)																					
Dagoretti	73/95	72.7	1.00				0.905														
Embakasi	102/146	66.1	0.73		0.37	1.43															
Kamukunji	12/18	64.3	0.68		0.20	2.32															
Kasarani	82/111	67.3	0.77		0.38	1.58															
Langata	23/34	73.3	1.03		0.37	2.87															
Makadara	15/18	79.9	1.49		0.30	7.48															
Starehe	64/83	74.3	1.08		0.49	2.40															
Westlands	41/53	76.5	1.22		0.48	3.08															
Outskirts	44/53	77.3	1.28		0.48	3.38															
Neighbourhood (Johannesburg)																					
Soweto												97/158	56.4	1.00			0.100	1.00			0.127
Hillbrow												30/44	60.7	1.19	0.53	2.68		0.94	0.34	2.55	
Brammfontein												23/28	77.6	2.67	0.72	9.89		1.90	0.50	7.20	
Orange Farm												12/13	94.7	13.74	1.63	115.50		13.60	1.39	132.44	
Other^a^												39/58	55.9	0.98	0.47	2.07		0.74	0.34	1.62	
Sexual identity																					
Gay	338/448	71.3	1.00				0.848					152/216	63.3	1.00			0.439				
Bisexual	108/143	73.4	1.11		0.67	1.83						41/71	53.2	0.66	0.35	1.26					
Heterosexual	0/2	0.0	–									0/3	0.0	–							
Other	5/9	65.0	0.75		0.16	3.46						6/9	55.4	0.72	0.13	3.98					
Gender Identity																					
Cisgender male	394/528	71.5	1.00				0.84					159/233	61.3	1.00			0.819				
Transfeminine	52/70	65.8	0.77		0.41	1.44						29/45	59.2	0.92	0.42	2.00					
Transmasculine	2/3	80.0	1.59		0.14	17.88						1/2	40.0	0.42	0.03	6.98					
Non–binary	13/17	71.0	0.98		0.26	3.65						12/21	51.1	0.66	0.23	1.92					
Monthly income (Nairobi)																					
<5000 KSH	165/224	72.3	1.00				0.572														
5000 to 9999 KSH	124/166	66.7	0.77		0.46	1.30															
10,000 to 19,999 KSH	103/129	75.5	1.18		0.65	2.15															
20,000 KSH +	40/55	69.7	0.88		0.41	1.92															
Monthly income (Johannesburg)																					
0 to 499 ZAR												56/82	63.1	1.00			0.930				
500 to 999 ZAR												25/39	57.6	0.80	0.32	1.97					
1000 to 1999 ZAR												40/57	61.1	0.92	0.39	2.19					
2000 to 4999 ZAR												48/74	58.2	0.82	0.37	1.81					
5000 + ZAR												21/30	68.3	1.26	0.43	3.69					
Employment status																					
Employed full‐time	42/57	67.9	0.84		0.40	1.79	0.255					20/32	45.4	0.58	0.23	1.48	0.486				
Employed part‐time	95/122	76.2	1.27		0.70	2.32						28/42	65.5	1.33	0.58	3.07					
Self‐employed	113/159	67.1	0.81		0.49	1.35						20/33	56.1	0.90	0.35	2.32					
Unemployed	187/247	71.5	1.00									110/168	58.7	1.00							
Student	11/12	95.4	8.34		1.00	69.59						16/19	75.9	2.22	0.54	9.11					
Other	7/11	59.1	0.58		0.12	2.78						6/6	100.0	–							
Completed educational attainment																					
Primary	69/111	58.8	0.47		0.28	0.80	0.022	0.40	0.23	0.70	0.006	1/3	66.7	1.38	0.12	15.81	0.892				
Secondary	256/329	75.1	1.00					1.00				146/222	59.1	1.00							
Higher Education	132/171	71.0	0.81		0.49	1.34		0.70	0.42	1.19		53/75	62.5	1.15	0.59	2.27					
Marital status																					
Not married	369/496	70.9	1.00				0.971					179/267	61.0	1.00			0.598				
Married to a man or transgender person	51/65	72.5	1.08		0.54	2.18						20/30	55.0	0.78	0.31	1.96					
Married to a woman	37/50	70.2	0.97		0.50	2.09						0/2	0.0	–							

Models weighted using RDS‐II weights (inverse network size) with seed participants dropped. Adjusted models include age a priori and those variables in bivariate analyses that showed an association of *p* < 0.1. aOR, adjusted odds ratio; KSH, Kenyan shillings (currency); MSM/TG, men who have sex with men and transgender people; OR, odds ratio; RDS, respondent‐driven sampling; ZAR, South African Rand (currency).

^a^ Other Johannesburg neighbourhoods include all those with fewer than 10 participants each.

There was no clear association between online socializing and age, nor sexual or gender identity, nor were there strong differences in the sociodemographic profiles of MSM and TG by use of gay‐specific or dating apps. (We show usage of sites apps separately by gender identity in Table S3 and Figures S2 and S3.) Those in the second lowest income category in Nairobi were more likely to use gay‐specific apps than those in the lowest, but there was no consistent trend. Those born outside of Kenya were more likely to use dating apps compared to MSM and TG born in Nairobi. Students in Johannesburg were more likely than others to use gay apps, and there was variation by neighbourhood, but there was no evidence for a difference by educational attainment or income, (Tables S4 and S5).

#### 3.1.5 Associations between sexual behaviour, STIs and online socializing

Evidence for associations between online socializing and sexual behaviour differed between cities (Table 3). In Nairobi, socializing with MSM online was more common among those who had: sex with a man in the last three months; receptive anal intercourse in the last three months; more sexual partners; and those who had either bought or sold sex to a man in the last 12 months. Socializing online was less common among those who had sex with a woman in the previous three months. Associations persisted after adjustment (Model 1) and when restricted to HIV‐uninfected participants (Model 2), though sex with a man in the last three months was not significant in the latter.

**Table 3:** Associations between socializing with MSM online in the previous month, recent sexual behaviours and sexually transmitted infection

Nairobi	Socialized online previous month		Crude, n = 608, without seeds				Adjusted, all participants (Model 1), n = 608, without seeds				Adjusted, HIV uninfected participants (Model 2), n = 424, without seeds			
	n	RDS %	OR	95% CI		*p* value	aOR	95% CI		*p* value	aOR	95% CI		*p* value
Overall socialized online with MSM in the last month	461/618	70.9												
Sex with a man (past three months)														
Yes	417/543	72.9	2.02	1.13	3.61	0.018	1.97	1.08	3.59	0.028	1.74	0.91	3.34	0.095
No	44/75	57.1	1.00				1.00				1.00			
Sex with a woman (past three months)														
Yes	116/174	63.5	0.62	0.40	0.96	0.032	0.61	0.38	0.96	0.032	0.55	0.33	0.94	0.029
No	345/444	73.8	1.00				1.00				1.00			
Condomless anal intercourse (past three months)														
Yes	212/265	75.5	1.48	0.97	2.28	0.072	1.37	0.88	2.14	0.161	1.67	0.97	2.87	0.063
No	249/353	67.6	1.00				1.00				1.00			
Receptive anal intercourse (past three months)														
Yes	202/321	76.6	1.72	1.13	2.62	0.011	1.69	1.10	2.60	0.017	2.15	1.25	3.69	0.006
No	259/321	65.5	1.00				1.00				1.00			
No. of male sexual partners (past three months)														
0	44/75	57.1	1.00			0.003	1.00			0.012	1.00			0.008
1	104/148	66.0	1.46	0.75	2.81		1.48	0.75	2.93		1.14	0.55	2.39	
2	121/171	69.9	1.75	0.91	3.34		1.72	0.88	3.36		1.65	0.78	3.50	
3 to 5	132/154	84.4	4.05	1.91	8.60		3.71	1.72	7.96		4.88	1.92	12.39	
6+	60/70	79.4	2.89	1.10	7.58		2.60	0.95	7.10		2.23	0.74	6.73	
Sold sex to a man (past 12 months)														
Yes	240/297	77.8	1.82	1.19	2.79	0.006	1.78	1.15	2.77	0.010	2.17	1.29	3.65	0.004
No	218/316	65.7	1.00				1.00				1.00			
Bought sex from a man (past 12 months)														
Yes	145/177	80.2	1.98	1.19	3.28	0.009	1.77	1.05	2.99	0.033	2.24	1.17	4.27	0.015
No	313/437	67.1	1.00				1.00				1.00			
STI symptoms (past 12 months)														
Yes	175/225	74.5	1.30	0.83	2.02	0.251	1.26	0.79	2.02	0.234	1.48	0.81	2.71	0.206
No	282/387	69.3	1.00				1.00				1.00			
Syphilis (active)														
Positive	5/5	100.0	–	–	–	–	–	–	–	–	–	–	–	–
Negative	454/609	70.7												
HIV														
Positive	146/186	76.2	1.43	0.89	2.32	0.134	1.37	0.81	2.31	0.243	–	–	–	–
Negative	314/431	69.0	1.00				1.00							

Johannesburg			Crude, n = 292, without seeds				Model 1, n = 292, without seeds				Model 2, n = 179, without seeds			
	n	RDS %	OR	95% CI		*p* value	aOR	95% CI		*p* value	aOR	95% CI		*p* value
Overall socialized online with MSM in the last month	201/301	60.1												
Sex with a man (past three months)														
Yes	163/234	62.1	1.42	0.74	2.74	0.292	1.32	0.66	2.64	0.431	1.72	0.76	3.91	0.194
No	38/67	53.6	1.00				1.00				1.00			
Sex with a woman (past three months)														
Yes	47/82	51.5	0.59	0.33	1.09	0.093	0.61	0.32	1.15	0.126	0.59	0.27	1.26	0.170
No	154/219	64.1	1.00				1.00				1.00			
Condomless anal intercourse (past three months)														
Yes	89/113	73.6	2.51	1.34	4.68	0.004	1.46	0.80	2.67	0.216	3.67	1.57	8.58	0.003
No	112/188	52.6	1.00				1.00				1.00			
Receptive anal intercourse (past three months)														
Yes	105/140	67.5	1.70	0.94	3.06	0.077	1.48	0.81	2.72	0.205	1.70	0.94	3.06	0.077
No	91/154	54.9	1.00				1.00				1.00			
No. of male sexual partners (past three months)														
0	38/67	53.6	1.00			0.192	1.00			0.301	1.00			0.323
1	61/94	55.8	1.09	0.52	2.30		1.03	0.47	2.26		1.37	0.55	3.46	
2	50/70	68.4	1.87	0.81	4.31		1.90	0.80	4.55		2.56	0.88	7.43	
3 to 5	36/52	61.9	1.41	0.56	3.56		1.16	0.44	3.05		1.42	0.40	5.06	
6+	16/18	86.5	5.54	1.07	28.63		5.39	0.71	41.02		11.49	0.50	266.40	
Sold sex to a man (past 12 months)														
Yes	47/69	60.1	0.97	0.50	1.91	0.941	0.92	0.45	1.88	0.809	0.87	0.34	2.25	0.777
No	152/228	60.7	1.00				1.00				1.00			
Bought sex from a man (past 12 months)														
Yes	17/31	39.4	0.38	0.16	0.93	0.036	0.35	0.14	0.85	0.022	0.31	0.09	1.03	0.056
No	182/266	63.0	1.00				1.00				1.00			
STI symptoms (past 12 months)														
Yes	61/89	60.7	1.07	0.57	2.00	0.840	1.11	0.57	2.16	0.766	0.83	0.35	1.97	0.672
No	137/209	59.2	1.00				1.00				1.00			
Syphilis (active)														
Positive	23/28	83.6	3.77	1.06	13.38	0.041	4.40	1.14	17.08	0.033	13.50	1.63	111.96	0.016
Negative	178/273	57.6	1.00				1.00				1.00			
HIV														
Positive	82/118	63.9	1.29	0.72	2.33	0.387	1.75	0.86	3.55	0.122	–	–	–	–
Negative	118/182	57.8	1.00				1.00							

Models weighted using RDS‐II weights (inverse network size) with seed participants dropped. Model 1: adjusted for age and sociodemographic characteristics found to be associated with online socializing in the previous month. Model 2: among only HIV uninfected participants, adjusted for age and sociodemographic characteristics found to be associated with online socializing in the previous month. aOR, adjusted odds ratio; KSH, Kenyan shillings (currency); MSM and TG, men who have sex with men and transgender people; OR, odds ratio; RDS, respondent‐driven sampling; ZAR, South African Rand (currency).

In Johannesburg, there was evidence for higher odds of online socializing among those reporting condomless anal intercourse in the last three months (crude model and among those HIV‐uninfected), but online socializing was significantly less often reported by participants who purchased sex from a man in the last 12 months, in both adjusted models. While the point estimates for associations between online socializing with men and having had sex with a man, sex with a woman and receptive anal intercourse in the previous three months among MSM and TG in Johannesburg were similar to those observed in Nairobi, the statistical evidence was weaker.

There was no significant difference in online socializing between the proportion of participants reporting STI symptoms or living with HIV by city. However, in Johannesburg, online socializing was associated with active syphilis.

Among MSM and TG who did report socializing online, those reporting some sexual behaviours associated with higher HIV transmission risks did have raised odds of using gay‐specific and dating‐specific apps, though not uniformly (Tables S6 and S7). In Nairobi, MSM and TG with more partners and who sold sex were more likely to report using gay‐specific apps, while in Johannesburg the association with selling sex was reversed (though the statistical evidence was somewhat weak). Use of dating apps was associated with both buying and selling sex in Nairobi, but not in Johannesburg. In both settings, socializing with MSM using dating apps was less common among MSM and TG who reported having had sex with a woman.

#### 3.1.6 Associations between online socializing and engagement with HIV prevention and care technologies

No significant associations were detected between online socializing in the last month and use of antiretroviral therapy (ART), virological suppression, recency of HIV testing, PrEP‐related knowledge (among those HIV uninfected or untested) or use or access to condoms or lubricants in either city (Table S1).

### 3.2 Qualitative findings

The sociodemographic characteristics of interview participants were consistent with those of the RDS samples in both cities. The majority (n = 54) of those interviewed reported regular use of social media to engage with other MSM and TG. Two clear themes were identified relevant to the delivery of interventions or research in online spaces.

#### 3.2.1 Navigating online environments

Participants commonly reported having multiple profiles on generic social media sites: one used for family or work friends and a second for engaging with other MSM. Much use was made of gay or MSM‐specific forums, groups and Facebook pages where participants reported how posting within such groups and searching their members for mutual friends was typically a reliable way of identifying other MSM. Posting a photo of oneself and seeing which men “liked” was considered a good initial strategy to online interaction.

*“There are those gay groups where you post your picture and say, ‘Please like me’ and you get like 50 messages in your inbox and like 20 friend requests in a day.” [Aged 20, Johannesburg]*

The use of gay‐specific apps was less common, though they enabled a more direct engagement with others known to be MSM. Positioning oneself in such an environment did, however, present its own risks which were often cited as the reason for using generic sites/apps. A number of interviewees also expressed the belief that gay‐specific apps remain the purview of MSM described as “higher class.” However, in both contexts, participants described using generic social media sites to find sexual partners, or to buy/sell sex.

Those with less sexual experience or confidence negotiating with men reported this setting as more amenable to flirtation and sexual‐planning. More common in both cities, however, was the use of social media to establish friendships, and to alleviate feelings of loneliness or isolation. A small number of men in both cities described how their first forays into MSM bars, clubs, hotels or other hot‐spots were facilitated by friendships originally made in online environments.

#### 3.2.2 Perceiving and mitigating harm

For many participants across both cities, interacting with men in social media environments was considered safer than attending physical locations where MSM congregate. This was especially true for participants in Kenya who reported that police raids or general hostility towards gay bars or hotspots were commonplace. However, while facilitating a broad range of social and sexual connectivity, social media apps and sites were not without their own risks.

Concerns were raised by participants in both cities regarding the possibility for “context collapse” [25]. This notion refers to when different aspects of one’s life and experience, which are usually kept separate in the physical world, come to overlap in online environments; for example, circumstances where a man’s family is unaware or disapproving of his attraction to men, this could be threatening. While having multiple profiles was a common strategy to avoid this occurrence, the risk remained. Particular concern was raised for pictures shared (especially sex‐related images) and how these might come to the attention of non‐MSM friends and family or be used for the purposes of blackmail. *“In social media the majority of them are looking for money. The others are blackmailers.”* [Aged 22, Nairobi]. Indeed, a concern for blackmail was pervasive among many, such that some were hesitant to share any personal information or images of themselves until they felt “safe” with the person(s) they were interacting with online. Ultimately choosing to meet face‐to‐face was challenged by these concerns. Risk mitigation strategies included “screening” the person online with questions about sex between men to ensure they were “legitimate” MSM, requesting that they send multiple photographs of themselves to ensure they had not simply adopted another person’s photograph), and actively discussing physical safety concerns before meeting.

*“Is it safe? Will people see me? Is there parking? Will there be people around?” [Aged 26, Nairobi]*

## 4 DISCUSSION

Use of social media to socialize with MSM was common among MSM and TG in Nairobi and Johannesburg, with a majority among population‐representative samples reporting having done so recently. Those socializing online showed a wide diversity of characteristics and clear HIV prevention and sexual health needs. MSM and TG used different platforms, but generic socializing apps/sites were most common, while use of gay‐specific and dating apps/sites were less prevalent, a pattern also seen amongst MSM seeking partners online in Nigeria [15]. All types of social media were used for partner‐seeking as well as socializing, but the role that social media plays in providing anonymity and security in the context of criminalizing and socially stigmatizing settings is complex.

Online socializing with MSM was not restricted to particular subgroups of socio‐economic status, age or sexual and gender identity. Nor was there good evidence that this varied very much by type of app, and the distribution of their use was very similar across cities. This is important because it suggests that a representative diversity of MSM and TG in each city might be reached via social media channels. There was some evidence that among MSM and TG in Nairobi online socializing was more common among those with at least a secondary education, compared to none or primary, but the use was high across all education groups. Although not directly comparable, our findings are unlike earlier surveys from Swaziland, Lesotho and Nigeria, in which online partner‐seeking was strongly associated with higher education and younger age [14, 15]. There was some evidence that social media use was higher amongst those who were born outside of Johannesburg, compared to those born in the city. This may reflect lower accessibility or tolerance of offline MSM communities outside of large cities, as reported in rural and semi‐rural parts of Mpumalanga, South Africa [22].

Our findings confirm that MSM and TG who are active online have unmet needs for sexual health information, services and referral. In both cities, MSM and TG socializing online reported high levels of HIV and STI transmission behaviours, yet we found no evidence they were more engaged in HIV prevention or care. The latter observation differs from studies in the United States suggesting that whilst Grindr users may engage in higher risk behaviours than non‐users, they also report higher uptake of prevention (PrEP use [37]) and HIV testing [7]). The delivery of HIV‐related education as well as signposting to HIV testing venues via engagement with MSM in social media (often termed “Netreach”) has been shown effective in several countries [38, 39], whereas social media facilitated HIV testing (e.g. for home‐based self‐testing or self‐sampling) has shown early promise as acceptable and feasible [40].

Effective and convenient sexual health promotion and service models facilitated by social media have been developed in high‐resource settings, and such models typically expand user choice beyond traditional, facility‐based services rather than replacing existing models completely [12]. Online interventions might help in mitigating the effects of healthcare associated stigma via facilitating better choice among services by enabling peer service reviews, for example [30, 41]. Site/app‐based channels of communication provide an additional option for peer support interventions, for instance, those aiming to improve ART adherence [42]. Importantly, our findings suggest that a sizeable minority of MSM and TG are not regularly active online (40% in Johannesburg, 30% in Nairobi). Some may not have access to the necessary technology, however, our qualitative findings indicate that for some, the risks of establishing a presence online are perceived to be greater than the benefits [24, 43]. Providers planning online services and interventions must be aware of and mitigate what may be unfamiliar risks, and should anticipate that such services will not be acceptable to all. Furthermore, the design of online interventions or intervention components need to consider the role of syndemics of mental health disorders, substance use and experience of harassment and abuse, which are part of the context in which sexual behaviours and risks among MSM and TG in Kenya and South Africa occur [44, 45, 46, 47], but which vary across settings [48]. A systematic review of substance use among African MSM found that use of recreational drugs and alcohol were frequently used as part of sexual experiences [49]. The ways in which these factors interact with the motivations for and experiences of online socializing and partner seeking among MSM and TG in Kenya and South Africa should be further explored to inform the targeting and design of intervention packages to address them.

We found some strong similarities in online socializing with MSM across the two cities, with similar types of site/apps used and concordance on the motivations for their use. Some differences in the associations with sexual behaviours may arise from differences in sample power, or different population prevalence of STIs (e.g. active syphilis is more prevalent in Johannesburg than Nairobi). There were different patterns associated with sexual exchange in each city, but this also varied substantially in prevalence; buying and selling of sex was twice as common among MSM and TG in Nairobi as in Johannesburg. The relative usefulness and strategies for using social media for engaging MSM and TG engaged in transactional sex might differ across cities.

A strength of our approach was the use of a representative survey method that permitted the comparison of those who are, and are not, socializing online (which cannot be accomplished in an online‐only survey). The qualitative data aid interpretation and make clear that, while not without its own challenges, online socializing helps facilitate in contexts of social censure. However, as a cross‐sectional survey, we are unable to determine causality relating to the experiences that influence social media use, nor infer direction of association between social media use and HIV transmission risk behaviours. Both study sites were urban and findings are not necessarily generalizable to rural areas. While widely used to obtain population‐representative estimates among MSM in SSA [40], assumptions underlying RDS estimation are difficult to meet in practice [51]. This survey lacked the power to explore issues specific to transgender participants and we acknowledge the need for such work. While there were lower percentages of transfeminine persons reporting use of gay‐specific and dating sites/apps in the last month, we did not find statistical evidence for differences in use of sites/apps to socialize with MSM in the last month by gender identity. Finally, the use of specific sites/apps changes quickly over time, but there is little more recent literature documenting and investigating online socializing among MSM and TG across SSA. In trying to understand motivations behind usage we hope that the data will be more enduringly informative.

## 5 CONCLUSIONS

Online environments are widely used by African MSM and TG to socialize and partner‐seek, and offer routes to deliver sexual health promotion, services and research opportunities that are currently under‐utilized. The lack of demographic variation in social media use suggests that access is not limited to those with higher socio‐economic status only. While evidence of an elevated HIV risk profile among those using social media may complicate the use of Internet‐facilitated samples to estimate HIV prevalence, MSM and TG recruited online may provide valuable insight into the factors that influence risk behaviour as well as uptake/engagement with HIV testing, care and prevention interventions. In addition, these data strengthen the evidence base for delivery of health education, social marketing and peer support programmes in online spaces. Since 2017, overall access to online sites/apps has likely increased and it is unlikely that the diversity of MSM and TG using them has narrowed. It is also possible that disruptions both to in‐person HIV and sexual health services, as well as to the options available for socializing amongst MSM and TG caused by the COVID‐19 pandemic and associated physical distancing policies further strengthen the need for online site/app mediated interventions. While needing to be attentive to concerns relating to context collapse and blackmail or digital security, community‐based organizations in particular are well‐placed to deliver online interventions at scale, potentially side‐stepping some of the traditional challenges inherent in reaching MSM and TG in hostile social environments that often present physical safety concerns.

## COMPETING INTEREST

No author has conflicts of interest to declare.

## AUTHORS’ CONTRIBUTIONS

EF contributed to conceiving and designing the study and data collection instruments, carried out quantitative analyses and drafting of the manuscript; AB contributed to conceiving and designing the study and data collection instruments, carried out qualitative analyses and drafting of the manuscript; ST and RK contributed to managing data collection, conducted interviews and approved the final draft; ADS contributed to designing the study, data collection instruments, carried out quantitative analyses, commented on and approved the final draft; WN helped design the data collection instruments, analysed qualitative data and approved the final draft; JK, PW and TPP contributed to conceiving and designing the study and data collection instruments, commented on the manuscript and approved the final draft. All authors have approved the final manuscript.

## Supporting information


**Figure S1.** Convergence of the estimate of the proportion of MSM/TG in Nairobi and Johannesburg who report having socialised online in the previous month.
**Figure S2.** Sites and App usage in Nairobi in the previous month by gender identity.
**Figure S3.** Sites and App usage in Johannesburg in the previous month by gender identity
**Table S1.** Associations between frequency of online socialising and engagement with HIV prevention and care technologies
**Table S2.** Demographic characteristics of qualitative interview participants
**Table S3.** Online socialising with MSM in the last month by gender identity
**Table S4.** Sociodemographic characteristics associated with socialising with MSM in the last month using gay‐specific apps, among MSM/TG who had socialised online in the last month
**Table S5.** Sociodemographic characteristics associated with socialising with MSM in the last month using dating‐specific apps, among MSM/TG who had socialised online in the last month
**Table S6.** Sexual behaviours and STIs associated with socialising with MSM in the last month using gay‐specific apps, among MSM/TG who had socialised online in the last month
**Table S7.** Sexual behaviours and STIs associated with socialising with MSM in the last month using dating‐specific apps, among MSM/TG who had socialised online in the last month
