# Supplementary material for: Online socializing among men who have sex with men and transgender people in Nairobi and Johannesburg and implications for public health‐related research and health promotion: an analysis of qualitative and respondent‐driven sampling survey data
Source: J Int AIDS Soc. 2020 Oct 1;23(Suppl 6):e25603. doi: 10.1002/jia2.25603 (PMC7527758; doi:10.1002/jia2.25603)
Supplement: Supplementary file 1 — Figure S1. Convergence of the estimate of the proportion of MSM/TG in Nairobi and Johannesburg who report having socialised online in the previous month. Figure S2. Sites and App usage in Nairobi in the previous month by gender identity. Figure S3. Sites and App usage in Johannesburg in the previous month by gender identity Table S1. Associations between frequency of online socialising and engagement with HIV prevention and care technologies Table S2. Demographic characteristics of qualitative interview participants Table S3. Online socialising with MSM in the last month by gender identity Table S4. Sociodemographic characteristics associated with socialising with MSM in the last month using gay‐specific apps, among MSM/TG who had socialised online in the last month Table S5. Sociodemographic characteristics associated with socialising with MSM in the last month using dating‐specific apps, among MSM/TG who had socialised online in the last month Table S6. Sexual behaviours and STIs associated with socialising with MSM in the last month using gay‐specific apps, among MSM/TG who had socialised online in the last month Table S7. Sexual behaviours and STIs associated with socialising with MSM in the last month using dating‐specific apps, among MSM/TG who had socialised online in the last month [file JIA2-23-e25603-s001.docx]

Supplementary Figure 1: Convergence of the estimate of the proportion of MSM/TG in Nairobi and Johannesburg who report having socialised online in the previous month

**A: Nairobi, n=618**

**B: Johannesburg, n=301**

The graphs show the cumulative RDS-II weighted proportion of MSM/TG (y axis) as the sample size increased (‘Observations’, x axis).

The estimate stabilised early in recruitment in Nairobi, and in Johannesburg stabilised well prior to recruitment ending. There was not an indication from these graphs that the estimate would have either increased or decreased had the sample size continued to increase.

Supplemental Table 1: Associations between frequency of online socialising and engagement with HIV and prevention and care technologies

|  |  | **Socialised online previous month** | |  | **Crude** | | | |  | **Adjusted** | | | |
| --- | --- | --- | --- | --- | --- | --- | --- | --- | --- | --- | --- | --- | --- |
| **Nairobi** |  | **n** | **RDS %** |  | **OR** | **95% CI** | | **p value** |  | **aOR** | **95% CI** | | **p value** |
|  |  |  |  |  |  |  |  |  |  |  |  |  |  |
| **HIV positive [n=186]** |  |  |  |  |  |  |  |  |  |  |  |  |  |
| Aware of HIV infection | Yes | 119/150 | 77.8 |  | 1.46 | 0.55 | 3.85 | 0.448 |  | 1.12 | 0.38 | 3.29 | 0.831 |
|  | No | 27/36 | 70.7 |  |  |  |  |  |  |  |  |  |  |
| Receiving anti-retroviral therapy | Yes | 101/129 | 77.3 |  | 1.20 | 0.50 | 2.87 | 0.688 |  | 0.94 | 0.34 | 2.58 | 0.903 |
|  | No | 45/57 | 74.0 |  |  |  |  |  |  |  |  |  |  |
| Virologically suppressed [<200 copies/ml) | Yes | 81/102 | 76.6 |  | 1.05 | 0.46 | 2.42 | 0.900 |  | 0.73 | 0.30 | 1.79 | 0.491 |
|  | No | 65/84 | 75.7 |  |  |  |  |  |  |  |  |  |  |
|  |  |  |  |  |  |  |  |  |  |  |  |  |  |
| **HIV uninfected [n=431]** |  |  |  |  |  |  |  |  |  |  |  |  |  |
| HIV test within last 6 months | Yes | 176/237 | 69.3 |  | 1.01 | 0.58 | 1.75 | 0.966 |  | 1.08 | 0.59 | 1.96 | 0.801 |
|  | No | 90/125 | 39.0 |  |  |  |  |  |  |  |  |  |  |
| Correct knowledge of PrEP | Yes | 156/208 | 71.3 |  | 1.28 | 0.79 | 2.09 | 0.319 |  | 1.25 | 0.74 | 2.11 | 0.408 |
|  | No | 144/217 | 65.9 |  |  |  |  |  |  |  |  |  |  |
| Current or previous use of PrEP | Yes | 34/43 | 71.0 |  | 1.11 | 0.45 | 2.76 | 0.820 |  | 1.17 | 0.45 | 3.06 | 0.750 |
|  | No | 271/377 | 68.7 |  |  |  |  |  |  |  |  |  |  |
| Correct knowledge of PEP | Yes | 156/208 | 71.1 |  | 1.18 | 0.7 | 1.93 | 0.504 |  | 1.09 | 0.64 | 1.85 | 0.743 |
|  | No | 150/210 | 67.6 |  |  |  |  |  |  |  |  |  |  |
| Previous use of PEP | Yes | 30/34 | 85.9 |  | 2.91 | 0.75 | 11.33 | 0.123 |  | 2.65 | 0.68 | 10.32 | 0.158 |
|  | No | 273/381 | 67.6 |  |  |  |  |  |  |  |  |  |  |
|  |  |  |  |  |  |  |  |  |  |  |  |  |  |
| **All participants [n=618]** |  |  |  |  |  |  |  |  |  |  |  |  |  |
| Problem accessing condoms when required | Yes | 188/255 | 70.3 |  | 0.92 | 0.60 | 1.41 | 0.71 |  | 0.93 | 0.60 | 1.46 | 0.766 |
|  | No | 261/345 | 72.0 |  |  |  |  |  |  |  |  |  |  |
| Problem accessing water-based lubricants when required | Yes | 235/320 | 70.1 |  | 0.91 | 0.60 | 1.39 | 0.672 |  | 0.94 | 0.61 | 1.46 | 0.786 |
|  | No | 214/281 | 72.0 |  |  |  |  |  |  |  |  |  |  |
|  |  |  |  |  |  | | | |  |  | | | |
|  |  |  |  |  |  | | | |  |  | | | |
|  |  |  |  |  |  | | | |  |  | | | |
|  |  |  |  |  |  | | | |  |  | | | |
|  |  |  |  |  |  | | | |  |  | | | |
|  |  |  |  |  |  | | | |  |  | | | |
|  |  |  |  |  |  | | | |  |  | | | |
|  |  | **Socialised online previous month** | |  | **Crude** | | | |  | **Adjusted** | | | |
| **Johannesburg** |  | **n** | **RDS %** |  | **OR** | **95% CI** | | **p value** |  | **aOR** | **95% CI** | | **p value** |
|  |  |  |  |  |  |  |  |  |  |  |  |  |  |
| **HIV positive [n=118]** |  |  |  |  |  |  |  |  |  |  |  |  |  |
| Aware of HIV infection | Yes | 50/76 | 57.0 |  | 0.50 | 0.19 | 1.37 | 0.179 |  | 0.67 | 0.22 | 2.01 | 0.474 |
|  | No | 31/41 | 72.4 |  |  |  |  |  |  |  |  |  |  |
| Receiving anti-retroviral therapy | Yes | 28/39 | 55.2 |  | 0.59 | 0.22 | 1.60 | 0.296 |  | 0.69 | 0.25 | 1.80 | 0.396 |
|  | No | 54/79 | 67.7 |  |  |  |  |  |  |  |  |  |  |
| Virologically suppressed [<200 copies/ml) | Yes | 40/64 | 53.2 |  | 0.39 | 0.15 | 1.04 | 0.059 |  | 0.40 | 0.15 | 1.10 | 0.076 |
|  | No | 42/54 | 74.4 |  |  |  |  |  |  |  |  |  |  |
|  |  |  |  |  |  |  |  |  |  |  |  |  |  |
| **HIV uninfected [n=182]** |  |  |  |  |  |  |  |  |  |  |  |  |  |
| HIV test within last 6 months | Yes | 65/96 | 58.8 |  | 1.12 | 0.49 | 2.57 | 0.784 |  | 1.05 | 0.44 | 2.49 | 0.914 |
|  | No | 30/50 | 56.0 |  |  |  |  |  |  |  |  |  |  |
| Correct knowledge of PrEP | Yes | 62/87 | 66.9 |  | 1.76 | 0.83 | 3.71 | 0.140 |  | 1.67 | 0.76 | 3.63 | 0.198 |
|  | No | 51/83 | 53.5 |  |  |  |  |  |  |  |  |  |  |
| Current or previous use of PrEP | Yes | 13/17 | 74.9 |  | 2.60 | 0.72 | 9.32 | 0.142 |  | 2.48 | 0.65 | 9.45 | 0.181 |
|  | No | 94/152 | 53.5 |  |  |  |  |  |  |  |  |  |  |
| Correct knowledge of PEP | Yes | 59/90 | 58.9 |  | 1.10 | 0.5 | 2.28 | 0.795 |  | 1.16 | 0.54 | 2.52 | 0.700 |
|  | No | 53/82 | 56.7 |  |  |  |  |  |  |  |  |  |  |
| Previous use of PEP | Yes | 11/14 | 79.7 |  | 3.19 | 0.76 | 13.43 | 0.112 |  | 3.28 | 0.79 | 13.50 | 0.100 |
|  | No | 98/157 | 55.1 |  |  |  |  |  |  |  |  |  |  |
|  |  |  |  |  |  |  |  |  |  |  |  |  |  |
| **All participants [n = 301]** |  |  |  |  |  |  |  |  |  |  |  |  |  |
| Problem accessing condoms when required | Yes | 85/135 | 56.3 |  | 0.76 | 0.43 | 1.34 | 0.335 |  | 0.81 | 0.45 | 1.45 | 0.470 |
|  | No | 110/159 | 63.0 |  |  |  |  |  |  |  |  |  |  |
| Problem accessing water-based lubricants when required | Yes | 62/146 | 57.6 |  | 0.80 | 0.45 | 1.42 | 0.440 |  | 0.83 | 0.46 | 1.48 | 0.520 |
|  | No | 103/147 | 63.0 |  |  |  |  |  |  |  |  |  |  |
| *Models weighted using RDS-II weights (inverse network size) with seed participants dropped.*  *Adjusted for age and sociodemographic characteristics found to be associated with online socialising* | | | | | |  |  |  |  |  |  |  |  |

Supplemental Table 2: Demographic characteristics of qualitative interview participants

| **Place of birth**  South Africa  Kenya  Elsewhere in Africa | **N**  28  25  7 | **Gender of partners in last 12 months**  Men only  Both men and women | **N**  47  13 | **Education level**  Primary  High school  College and above | **N**  6  32  22 |
| --- | --- | --- | --- | --- | --- |
| **HIV status**  Diagnosed positive  HIV negative  Never tested  **Gender Identity**  Transfeminine  Cisgender | **N**  13  1  46  2  58 | **Age**  Mean  SD | **N**  30.5  8.7 | **Relationship status**  Married  Long term but not married  Newly initiated relationship  Single | **N**  5  27  6  22 |

Supplemental Figure 2: Sites and App usage in Nairobi in the previous month by gender identity

*Transmasculine participants (n=3) not shown here due to privacy concerns.*

Supplemental Figure 3: Sites and App usage in Johannesburg in the previous month by gender identity

*Transmasculine participants (n=2) not shown here due to privacy concerns.*

Supplemental Table 3: Online socialising with MSM in the last month by gender identity

| **Johannesburg** | |  |  |  |  |  |  |  |  |  |  |  |  |
| --- | --- | --- | --- | --- | --- | --- | --- | --- | --- | --- | --- | --- | --- |
| **Socialising online with MSM in the previous month:** | | | | |  |  |  |  |  |  |  |  |  |
|  |  | **n** | **%** | **RDS%** |  |  |  |  |  |  |  |  |  |
| Cisgender |  | 159/233 | 68.2 | 61.3 |  |  |  |  |  |  |  |  |  |
| Transfeminine | | 29/45 | 64.4 | 59.2 |  |  |  |  |  |  |  |  |  |
| Non-binary |  | 12/21 | 57.1 | 51.1 |  |  |  |  |  |  |  |  |  |
|  | **Of these:** | **Generic** | | |  | **Gay** | | |  | **Dating** | | |  |
|  |  | **n** | **%** | **RDS%** |  | **n** | **%** | **RDS%** |  | **n** | **%** | **RDS%** |  |
| Cisgender |  | 139/159 | 87.4 | 87 |  | 62/159 | 39.0 | 33.2 |  | 30/159 | 18.9 | 20.3 |  |
| Transfeminine | | 25/29 | 86.2 | 85.1 |  | 7/29 | 24.1 | 19.6 |  | 4/29 | 13.8 | 8.8 |  |
| Non-binary |  | 12/12/ | 100.0 | 100 |  | 2/12 | 16.7 | 15.7 |  | 3/12 | 25.0 | 25.1 |  |
|  |  |  |  |  |  |  |  |  |  |  |  |  |  |
|  |  |  |  |  |  |  |  |  |  |  |  |  |  |
| **Nairobi** |  |  |  |  |  |  |  |  |  |  |  |  |  |
| **Socialising online with MSM in the previous month:** | | | | |  |  |  |  |  |  |  |  |  |
|  |  | **n** | **%** | **RDS%** |  |  |  |  |  |  |  |  |  |
| Cisgender |  | 394/528 | 74.6 | 71.5 |  |  |  |  |  |  |  |  |  |
| Transfeminine | | 52/70 | 74.3 | 65.8 |  |  |  |  |  |  |  |  |  |
| Non-binary |  | 13/17 | 76.5 | 71 |  |  |  |  |  |  |  |  |  |
|  | **Of these:** | **Generic** | | |  | **Gay** | | |  | **Dating** | | |  |
|  |  | **n** | **%** | **RDS%** |  | **n** | **%** | **RDS%** |  | **n** | **%** | **RDS%** |  |
| Cisgender |  | 359/394 | 91.1 | 92.3 |  | 148/394 | 37.6 | 31.1 |  | 60/394 | 15.2 | 12.0 |  |
| Transfeminine | | 47/52 | 90.4 | 92.3 |  | 16/52 | 30.8 | 31.8 |  | 6/52 | 11.5 | 7.4 |  |
| Non-binary |  | 13/13 | 100.0 | 100 |  | 6/13 | 46.2 | 30.5 |  | 3/13 | 23.1 | 27.0 |  |
|  |  |  |  |  |  |  |  |  |  |  |  |  |  |
| *Transmasculine participants (n=2 in Johannesburg and n=3 in Nairobi) not shown here for concern to privacy and small numbers in the study sample.* | | | | | | | | | | | | | |
| *Note: We do not find statistical evidence from logistic regression models, supplemental tables 4 and 5,* | | | | | | | | | | | | | |
| *for an association between type of app/site used in the previous month and gender identity.* | | | | | | | | | | |  |  |  |

Supplemental Table 4: Sociodemographic characteristics associated with socialising with MSM in the last month using gay-specific apps, among MSM/TG who had socialised online in the last month

|  |  | **Socialised using Gay-specific apps** | |  | **Crude** | | | |
| --- | --- | --- | --- | --- | --- | --- | --- | --- |
| **Nairobi, n=461 MSM/TG who have socialised online in the previous month** | | **n** | **RDS %** |  | **OR** | **95% CI** | | **p value** |
| Overall socialising online using gay-specific apps in the previous month | | **171/461** | **31.2** |  |  |  |  |  |
| Age group | 18-21 | 44/112 | 35.4 |  | 1.00 |  |  | 0.641 |
|  | 22-24 | 43/134 | 29.0 |  | 0.75 | 0.40 | 1.38 |  |
|  | 25-29 | 50/111 | 33.2 |  | 0.91 | 0.48 | 1.72 |  |
|  | 30+ | 34/104 | 27.2 |  | 0.68 | 0.35 | 1.32 |  |
| Born in Nairobi | Born in Nairobi | 47/124 | 35.0 |  | 1.00 |  |  | 0.347 |
|  | Born elsewhere in Kenya | 82/226 | 27.8 |  | 0.72 | 0.42 | 1.22 |  |
|  | Born outside Kenya | 39/98 | 35.3 |  | 1.01 | 0.53 | 1.93 |  |
| Religion | Christianity | 151/404 | 31.5 |  | 1.00 | 1.00 | 1.00 | 0.541 |
|  | Islam | 14/42 | 27.5 |  | 0.82 | 0.35 | 1.95 |  |
|  | Other or none | 4/11 | 16.3 |  |  |  |  |  |
|  |  |  |  |  | 0.42 | 0.08 | 2.16 |  |
| Neighbourhood | Dagoretti | 27/73 | 33.4 |  | 1.00 |  |  | 0.493 |
|  | Embakasi | 41/102 | 33.5 |  | 1.01 | 0.48 | 2.10 |  |
|  | Kamukunji | 4/12 | 27.0 |  | 0.74 | 0.15 | 3.58 |  |
|  | Kasarani | 25/82 | 19.6 |  | 0.49 | 0.22 | 1.07 |  |
|  | Langata | 9/23 | 28.2 |  | 0.78 | 0.25 | 2.46 |  |
|  | Makadara | **7/15** | **43.1** |  | 1.51 | 0.39 | 5.88 |  |
|  | Starehe | **21/64** | **30.6** |  | 0.88 | 0.38 | 2.04 |  |
|  | Westlands | 16/41 | 41.9 |  | 1.44 | 0.58 | 3.57 |  |
|  | Outskirts | 18/44 | 31.7 |  | 0.92 | 0.37 | 2.33 |  |
|  | Missing | 3/5 | 54.3 |  | 2.37 | 0.28 | 19.88 |  |
| Sexual identity | Gay | 126/338 | 30.8 |  | 1.00 |  |  | 0.960 |
|  | Bisexual | 39/108 | 32.3 |  | 1.07 | 0.63 | 1.83 |  |
|  | Heterosexual | 0/0 |  |  | - | - | - |  |
|  | Other | 4/10 | 29.1 |  | 0.92 | 0.17 | 5.02 |  |
| Gender Identity | Cisgender male | 148/394 | 31.1 |  | 1.00 |  |  | 0.956 |
|  | Transfeminine | 16/52 | 31.8 |  | 1.03 | 0.50 | 2.15 |  |
|  | Transmasculine | 1/2 | 50.0 |  | 2.22 | 0.14 | 35.98 |  |
|  | Non-binary | 6/13 | 30.5 |  | 0.97 | 0.21 | 4.48 |  |
| Monthly income | <5000 KSH | 49/165 | 25.8 |  | 1.00 |  |  | 0.023 |
|  | 5000-9999 KSH | 55/124 | 41.4 |  | 2.03 | 1.13 | 3.64 |  |
|  | 10,000-19,999 KSH | 43/103 | 37.3 |  | 1.71 | 0.93 | 3.14 |  |
|  | 20,000 KSH + | 15/40 | 16.0 |  | 0.55 | 0.21 | 1.41 |  |
|  | Missing | 9/29 | 25.6 |  | 0.99 | 0.37 | 2.62 |  |
| Employment status | Employed full-time | 14/42 | 28.5 |  | 1.00 |  |  | 0.097 |
|  | Employed part-time | 50/95 | 43.9 |  | 1.96 | 0.80 | 4.82 |  |
|  | Self-employed | 44/113 | 33.6 |  | 1.27 | 0.53 | 3.07 |  |
|  | Unemployed | *54/187* | *23.5* |  | 0.77 | 0.33 | 1.80 |  |
|  | Student | *3/11* | *28.7* |  | 1.01 | 0.17 | 6.12 |  |
|  | Other | 3/7 | 32.5 |  | 1.21 | 0.17 | 8.38 |  |
| Completed Educational Attainment | Primary | 17/69 | 27.1 |  | 1.23 | 0.29 | 5.21 | 0.871 |
|  | Secondary | 86/156 | 26.6 |  | 1.00 |  |  |  |
|  | Higher Education | 66/132 | 42.8 |  | 1.23 | 0.53 | 2.86 |  |
| Marital Status | Not married | 142/369 | 32.1 |  | 1.00 |  |  | 0.436 |
|  | Married to a man or transgender person | 17/51 | 30.6 |  | 0.93 | 0.44 | 1.99 |  |
|  | Married to a woman | 10/37 | 20.8 |  | 0.56 | 0.23 | 1.36 |  |
|  |  |  |  |  |  |  |  |  |
| **Johannesburg, n=201 MSM/TG who have socialised online in the previous month** | | **n** | **RDS %** |  | **OR** | **95% CI** | | **p value** |
| Overall socialising online using gay-specific apps in the previous month | | 71/201 | 30.2 |  |  |  |  |  |
| Age group | 18-21 | 20/55 | 34.1 |  | 1.00 |  |  | 0.826 |
|  | 22-24 | 17/47 | 32.9 |  | 0.95 | 0.35 | 2.55 |  |
|  | 25-29 | 15/48 | 24.4 |  | 0.63 | 0.22 | 1.79 |  |
|  | 30+ | 19/51 | 29.2 |  | 0.80 | 0.29 | 2.16 |  |
| Born in Johannesburg | Born in Johannesburg | 36/113 | 28.7 |  | 1.00 |  |  | 0.718 |
|  | Born elsewhere in South Africa | 29/75 | 30.8 |  | 1.10 | 0.52 | 2.36 |  |
|  | Born outside South Africa | 6/12 | 43.1 |  | 1.88 | 0.41 | 8.57 |  |
| Religion | Religious (all but 2 Christianity) | 67/177 | 33.5 |  | 1.00 |  |  | 0.066 |
|  | Not religious | 4/24 | 11.2 |  | 0.25 | 0.06 | 1.09 |  |
| Neighbourhood | Soweto | 32/97 | 28.7 |  | 1.00 |  |  | <0.001 |
|  | Hillbrow | 7/30 | 20.8 |  | 0.62 | 0.20 | 1.90 |  |
|  | Brammfontein | 16/23 | 74.9 |  | 7.35 | 1.97 | 27.40 |  |
|  | Orange Farm | 1/12 | 0.4 |  | 0.01 | 0.00 | 0.08 |  |
|  | Other* | 15/39 | 0.24 |  | 1.04 | 0.40 | 2.72 |  |
| Sexual identity | Gay or homosexual | 59/152 | 32.7 |  | 1.00 |  |  | 0.483 |
|  | Bisexual | 9/41 | 22.2 |  | 0.59 | 0.22 | 1.54 |  |
|  | Heterosexual | 0/0 | - |  | - | - | - |  |
|  | Other | 3/6 | 43.7 |  | 1.60 | 0.20 | 12.77 |  |
| Gender Identity | Cisgender male | 62/159 | 33.2 |  | 1.00 |  |  | 0.314 |
|  | Transfeminine | 7/29 | 19.6 |  | 0.51 | 0.18 | 1.44 |  |
|  | Transmasculine | 0/1 | 0 |  | - | - | - |  |
|  | Non-binary | 2/12 | 15.7 |  | 0.37 | 0.05 | 2.96 |  |
| Monthly income | 0-499 ZAR | 22/56 | 38 |  | 1.00 |  |  | 0.106 |
|  | 500-999 ZAR | 7/25 | 27.6 |  | 0.58 | 0.17 | 1.95 |  |
|  | 1000-1999 ZAR | 20/40 | 45.2 |  | 1.25 | 0.44 | 3.49 |  |
|  | 2000-4999 ZAR | 15/48 | 18.4 |  | 0.34 | 0.13 | 0.89 |  |
|  | 5000+ ZAR | 6/21 | 24.6 |  | 0.49 | 0.12 | 2.08 |  |
| Employment status | Employed full-time | 6/20 | 26 |  | 1.00 |  |  | 0.009 |
|  | Employed part-time | 7/28 | 20.7 |  | 0.74 | 0.15 | 3.71 |  |
|  | Self-employed | 5/20 | 19.9 |  | 0.71 | 0.11 | 4.57 |  |
|  | Unemployed | 37/110 | 28.6 |  | 1.14 | 0.31 | 4.21 |  |
|  | Student | 14/16 | 89.4 |  | 24.15 | 3.39 | 172.15 |  |
|  | Other | 2/6 | 25.7 |  | 0.99 | 0.11 | 8.86 |  |
| Completed Educational Attainment | Primary | 5/14 | 29 |  | 1.23 | 0.29 | 5.21 | 0.871 |
|  | Secondary | 49/133 | 33.5 |  | 1.00 |  |  |  |
|  | Higher Education | s | 33.4 |  | 1.23 | 0.53 | 2.86 |  |
| Marital Status | Not married | 60/179 | 28.3 |  | 1.00 |  |  | 0.173 |
|  | Married to a man or transgender person | 10/20 | 46.7 |  | 2.22 | 0.71 | 6.92 |  |
|  | Married to a woman | 0/0 | - |  | - | - | - |  |
|  |  |  |  |  |  |  |  |  |
| *neighbourhoods with fewer than 10 participants | |  |  |  |  |  |  |  |

Supplemental Table 5: Sociodemographic characteristics associated with socialising with MSM in the last month using dating-specific apps, among MSM/TG who had socialised online in the last month

|  |  | **Socialised using Dating-specific apps** | |  | **Crude** | | | |
| --- | --- | --- | --- | --- | --- | --- | --- | --- |
| **Nairobi, n=461 MSM/TG who have socialised online in the previous month** | | **n** | **RDS %** |  | **OR** | **95% CI** | | **p value** |
| Overall socialising online using dating-specific apps in the previous month | | 69/461 | 11.8 |  |  |  |  |  |
| Age group | 18-21 | 10/112 | 9.0 |  | 1.00 |  |  | 0.688 |
|  | 22-24 | 19/134 | 11.1 |  | 1.26 | 0.49 | 3.23 |  |
|  | 25-29 | 25/111 | 14.3 |  | 1.70 | 0.67 | 4.28 |  |
|  | 30+ | 15/104 | 13.4 |  | 1.56 | 0.58 | 4.23 |  |
| Born in Nairobi | Born in Nairobi | 16/124 | 8.6 |  | 1.00 |  |  | 0.019 |
|  | Born elsewhere in Kenya | 25/226 | 9.8 |  | 1.16 | 0.51 | 2.66 |  |
|  | Born outside Kenya | 27/98 | 21.3 |  | 2.88 | 1.23 | 6.76 |  |
| Religion | Christianity | 55/404 | 11.4 |  | 1.00 |  |  | 0.650 |
|  | Islam | 9/42 | 5.2 |  | 1.40 | 0.50 | 3.92 |  |
|  | Other or none | 4/11 | 18.5 |  | 1.77 | 0.37 | 8.33 |  |
| Neighbourhood | Dagoretti | 16/73 | 16.0 |  | 1.00 |  |  | 0.543 |
|  | Embakasi | 12/102 | 10.6 |  | 0.62 | 0.22 | 1.73 |  |
|  | Kamukunji | 1/12 | 1.3 |  | 0.07 | 0.01 | 0.59 |  |
|  | Kasarani | 13/82 | 11.8 |  | 0.70 | 0.26 | 1.92 |  |
|  | Langata | 3/23 | 10.7 |  | 0.63 | 0.12 | 3.25 |  |
|  | Makadara | **2/15** | **15.9** |  | 0.99 | 0.18 | 5.37 |  |
|  | Starehe | **8/64** | **9.5** |  | 0.55 | 0.18 | 1.71 |  |
|  | Westlands | 7/41 | 16.6 |  | 1.04 | 0.34 | 3.23 |  |
|  | Outskirts | 6/44 | 8.3 |  | 0.48 | 0.15 | 1.53 |  |
|  | Missing | 1/5 | 11.4 |  | 0.68 | 0.06 | 7.17 |  |
| Sexual identity | Gay | 51/338 | 11.3 |  | 1.00 |  |  | 0.473 |
|  | Bisexual | 15/108 | 11.9 |  | 1.05 | 0.50 | 2.21 |  |
|  | Heterosexual | 0 |  |  |  |  |  |  |
|  | Other | 2/10 | 27.7 |  | 3.00 | 0.52 | 17.42 |  |
| Gender Identity | Cisgender male | 60/394 | 12.0 |  | 1.00 |  |  | 0.275 |
|  | Transfeminine | 6/52 | 7.4 |  | 0.59 | 0.21 | 1.65 |  |
|  | Transmasculine | 0/2 |  |  | - | - | - |  |
|  | Non-binary | 3/13 | 27.0 |  | 2.72 | 0.52 | 14.19 |  |
| Monthly income | <5000 KSH | 21/165 | 8.7 |  | 1.00 |  |  | 0.181 |
|  | 5000-9999 KSH | 19/124 | 15.3 |  | 1.89 | 0.84 | 4.25 |  |
|  | 10,000-19,999 KSH | 19/103 | 15.9 |  | 1.98 | 0.87 | 4.51 |  |
|  | 20,000 KSH + | 5/40 | 4.6 |  | 0.50 | 0.13 | 1.96 |  |
|  | Missing | 5/29 | 12.2 |  | 1.45 | 0.41 | 5.15 |  |
| Employment status | Employed full-time | 4/42 | 8.1 |  | 1.00 |  |  | 0.065 |
|  | Employed part-time | 23/95 | 19.4 |  | 2.75 | 0.65 | 11.65 |  |
|  | Self-employed | 17/113 | 14.2 |  | 1.89 | 0.44 | 8.10 |  |
|  | Unemployed | *23/187* | 8.0 |  | 0.99 | 0.24 | 4.12 |  |
|  | Student | *0/11* | *-* |  | - | - | - |  |
|  | Other | 0/7 | - |  | - | - | - |  |
| Completed Educational Attainment | Primary | 9/69 | 11.2 |  | 1.27 | 0.48 | 3.33 | 0.082 |
|  | Secondary | 29/256 | 9.1 |  |  |  |  |  |
|  | Higher Education | 30/132 | 17.9 |  | 2.18 | 1.10 | 4.34 |  |
| Marital Status | Not married | 54/369 | 11.1 |  | 1.00 |  |  | 0.552 |
|  | Married to a man or transgender person | 8/51 | 17.2 |  | 1.68 | 0.65 | 4.34 |  |
|  | Married to a woman | 6/37 | 13.3 |  | 1.24 | 0.40 | 3.81 |  |
|  |  |  |  |  |  |  |  |  |
| **Johannesburg, n=201 MSM/TG who have socialised online in the previous month** | | **n** | **RDS %** |  | **OR** | **95% CI** | | **p value** |
| Overall socialising online using dating-specific apps in the previous month | | 37/201 | 19.1 |  |  |  |  |  |
| Age group | 18-21 | 11/55 | 23.4 |  | 1.00 |  |  | 0.068 |
|  | 22-24 | 2/47 | 3.8 |  | 0.13 | 0.02 | 0.68 |  |
|  | 25-29 | 12/48 | 21.5 |  | 0.90 | 0.28 | 2.88 |  |
|  | 30+ | 12/51 | 26.4 |  | 1.18 | 0.38 | 3.64 |  |
| Born in Johannesburg | Born in Johannesburg | 22/113 | 20 |  | 1.00 |  |  | 0.968 |
|  | Born elsewhere in South Africa | 13/75 | 18.2 |  | 0.89 | 0.35 | 2.24 |  |
|  | Born outside South Africa | 2/12 | 18.5 |  | 0.91 | 0.1 | 8.06 |  |
| Religion | Religious (all but 2 Christianity) | 34/177 | 21.6 |  | 1.00 |  |  | 0.030 |
|  | Not religious | 3/24 | 4.7 |  | 0.18 | 0.04 | 0.84 |  |
| Neighbourhood | Soweto | 19/97 | 20.8 |  | 1.00 |  |  | 0.866 |
|  | Hillbrow | 5/30 | 13.4 |  | 0.64 | 0.16 | 2.51 |  |
|  | Brammfontein | 4/23 | 18.6 |  | 0.98 | 0.21 | 4.55 |  |
|  | Orange Farm | 2/12 | 20.3 |  | 1.10 | 0.16 | 7.64 |  |
|  | Other* | 7/39 | 29 |  | 1.56 | 0.48 | 5.05 |  |
| Sexual identity | Gay | 32/152 | 22.6 |  | 1.00 |  |  | 0.238 |
|  | Bisexual | 4/41 | 8.4 |  | 0.32 | 0.08 | 1.2 |  |
|  | Heterosexual | 0/0 | - |  | - | - | - |  |
|  | Other | 1/6 | 23 |  | 1.02 | 0.09 | 11.02 |  |
| Gender Identity | Cisgender male | 30/159 | 20.3 |  | 1.00 |  |  | 0.355 |
|  | Transfeminine | 4/29 | 8.8 |  | 0.39 | 0.1 | 1.52 |  |
|  | Transmasculine | 0/1 | 0 |  | - | - |  |  |
|  | Non-binary | 3/12 | 25.1 |  | 1.32 | 0.26 | 6.64 |  |
| Monthly income | 0-499 ZAR | 8/56 | 21.7 |  | 1.00 |  |  | 0.795 |
|  | 500-999 ZAR | 3/25 | 11.5 |  | 0.46 | 0.09 | 2.34 |  |
|  | 1000-1999 ZAR | 9/40 | 18.8 |  | 0.83 | 0.24 | 2.92 |  |
|  | 2000-4999 ZAR | 10/48 | 22.2 |  | 1.03 | 0.3 | 3.48 |  |
|  | 5000+ ZAR | 7/21 | 29.1 |  | 1.48 | 0.34 | 6.35 |  |
| Employment status | Employed full-time | 3/20 | 12 |  | 1.00 |  |  | 0.148 |
|  | Employed part-time | 7/28 | 15.4 |  | 1.33 | 0.19 | 9.17 |  |
|  | Self-employed | 3/20 | 4.3 |  | 0.33 | 0.04 | 2.98 |  |
|  | Unemployed | 18/110 | 20.9 |  | 1.93 | 0.36 | 10.55 |  |
|  | Student | 5/16 | 42.5 |  | 5.44 | 0.69 | 42.97 |  |
|  | Other | 1/6 | 17.1 |  | 1.52 | 0.1 | 23.13 |  |
| Completed Educational Attainment | Primary | 2/14 | 9.8 |  | 0.56 | 0.1 | 2.97 | 0.151 |
|  | Secondary | 21/133 | 16.3 |  | 1.00 |  |  |  |
|  | Higher Education | 14/53 | 30.11 |  | 2.22 | 0.86 | 5.72 |  |
| Marital Status | Not married | 34/179 | 19.6 |  | 1.00 |  |  | 0.801 |
|  | Married to a man or transgender person | 3/120 | 16.6 |  | 0.82 | 0.18 | 3.81 |  |
|  | Married to a woman | 0/0 | - |  | - | - | - |  |
|  |  |  |  |  |  |  |  |  |
| *neighbourhoods with fewer than 10 participants | |  |  |  |  |  |  |  |

Supplemental Table 6: Sexual behaviours and STIs associated with socialising with MSM in the last month using gay-specific apps, among MSM/TG who had socialised online in the last month

|  |  | **Socialised using Gay-Specific apps** | |  | **Crude** | | | |  | **Adjusted for income and employment** | | | |
| --- | --- | --- | --- | --- | --- | --- | --- | --- | --- | --- | --- | --- | --- |
| **Nairobi, n=461 MSM/TG who have socialised online in the previous month** |  | **n** | **RDS %** |  | **OR** | **95% CI** | | **p value** |  | **aOR** | **95% CI** | | **p value** |
| Overall socialising online using gay-specific apps in the previous month |  | 171/461 | 31.2 |  |  |  |  |  |  |  |  |  |  |
|  |  |  |  |  |  |  |  |  |  |  |  |  |  |
| Sex with a man in the past 3 months |  | 158/417 | 31.9 |  | 1.37 | 0.62 | 3.02 | 0.437 |  | 1.18 | 0.54 | 2.59 | 0.677 |
| Sex with a woman in the past 3 months |  | 41/116 | 33.2 |  | 1.13 | 0.67 | 1.91 | 0.639 |  | 1.04 | 0.59 | 1.81 | 0.898 |
| Condomless anal intercourse (3 months) |  | 72/212 | 28.5 |  | 0.80 | 0.50 | 1.26 | 0.328 |  | 0.79 | 0.49 | 1.27 | 0.330 |
| Receptive anal sex (3 months) |  | 110/259 | 34.8 |  | 1.44 | 0.91 | 2.28 | 0.123 |  | 1.33 | 0.83 | 2.15 | 0.238 |
| Number of sexual partners (3 months) | 0 | 13/44 | 25.4 |  | 1.00 |  |  | <0.001 |  |  |  |  | 0.001 |
|  | 1 | 18/104 | 14.1 |  | 0.48 | 0.18 | 1.26 |  |  | 0.41 | 0.16 | 1.09 |  |
|  | 2 | 44/121 | 34.0 |  | 1.51 | 0.64 | 3.57 |  |  | 1.37 | 0.58 | 3.22 |  |
|  | 3-5 | 60/132 | 40.7 |  | 2.01 | 0.85 | 4.75 |  |  | 1.57 | 0.67 | 3.68 |  |
|  | 6+ | 36/60 | 54.2 |  | 3.47 | 1.24 | 9.69 |  |  | 3.27 | 1.19 | 9.01 |  |
| Sold sex to a man (12 months) |  | 106/240 | 36.7 |  | 1.66 | 1.05 | 2.62 | 0.031 |  | 1.84 | 1.14 | 2.97 | 0.012 |
| Bought sex from a man (12 months) |  | 58/145 | 34.4 |  | 1.25 | 0.77 | 2.04 | 0.368 |  | 1.29 | 0.78 | 2.11 | 0.320 |
| STI symptoms (12 months) |  | 75/175 | 36.2 |  | 1.44 | 0.91 | 2.29 | 0.123 |  | 1.34 | 0.83 | 2.17 | 0.225 |
| CT/NG (rectal or urethral) |  | 55/123 | 39.3 |  | 1.64 | 1.00 | 2.71 | 0.052 |  | 1.64 | 0.97 | 2.76 | 0.065 |
| Syphilis (active) |  | 3/5 | 65.2 |  | 2.73 | 0.29 | 26.10 | 0.382 |  | 4.42 | 0.50 | 39.39 | 0.184 |
|  |  |  |  |  |  |  |  |  |  |  |  |  |  |
|  |  | **Socialised using Gay-Specific apps** | |  | **Crude** | | | |  | **Adjusted for religion, employment and neighbourhood** | | | |
| **Johannesburg, n=201 MSM/TG who have socialised online in the previous month** | | **n** | **RDS %** |  | **OR** | **95% CI** | | **p value** |  | **aOR** | **95% CI** | | **p value** |
| Overall socialising online using gay-specific apps in the previous month |  | 71/201 | 30.2 |  |  |  |  |  |  |  |  |  |  |
|  |  |  |  |  |  |  |  |  |  |  |  |  |  |
| Sex with a man in the past 3 months |  | 58/163 | 29.9 |  | 0.94 | 0.37 | 2.40 | 0.892 |  | 1.30 | 0.46 | 3.69 | 0.625 |
| Sex with a woman in the past 3 months |  | 10/47 | 18.9 |  | 0.44 | 0.17 | 1.14 | 0.094 |  | 0.49 | 0.17 | 1.39 | 0.181 |
| Condomless anal intercourse (3 months) |  | 36/89 | 38.3 |  | 1.99 | 0.96 | 4.15 | 0.068 |  | 1.95 | 0.83 | 4.56 | 0.126 |
| Receptive anal sex (3 months) |  | 42/105 | 32.3 |  | 1.21 | 0.59 | 2.52 | 0.604 |  | 1.40 | 0.62 | 3.17 | 0.414 |
| Number of sexual partners (3 months) | 0 | 13/38 | 31.3 |  |  |  |  | 0.392 |  | 1.00 |  |  | 0.317 |
|  | 1 | 19/61 | 24.7 |  | 0.72 | 0.24 | 2.13 |  |  | 0.98 | 0.29 | 3.39 |  |
|  | 2 | 13/50 | 25.8 |  | 0.77 | 0.24 | 2.40 |  |  | 1.07 | 0.31 | 3.62 |  |
|  | 3-5 | 15/36 | 40.1 |  | 1.47 | 0.44 | 4.95 |  |  | 2.63 | 0.71 | 9.84 |  |
|  | 6+ | 11/16 | 52.5 |  | 2.43 | 0.54 | 10.86 |  |  | 3.56 | 0.55 | 23.19 |  |
| Sold sex to a man (12 months) |  | 14/47 | 17.9 |  | 0.43 | 0.18 | 1.02 | 0.056 |  | 0.42 | 0.17 | 1.06 | 0.067 |
| Bought sex from a man (12 months) |  | 6/17 | 22.9 |  | 0.67 | 0.19 | 2.39 | 0.541 |  | 0.65 | 0.16 | 2.68 | 0.555 |
| STI symptoms (12 months) |  | 29/61 | 42.8 |  | 2.24 | 1.02 | 4.89 | 0.045 |  | 3.32 | 1.38 | 7.96 | 0.008 |
| Syphilis (active) |  | 6/23 | 26.7 |  | 0.82 | 0.26 | 2.59 | 0.739 |  | 0.67 | 0.19 | 2.37 | 0.535 |
| CT (urethral only) |  | 1/8 | 29.1 |  | 0.95 | 0.10 | 8.81 | 0.964 |  | 1.12 | 0.12 | 10.42 | 0.919 |
| NG (urethral only) |  | 1/3 | 13.0 |  | 0.34 | 0.03 | 4.31 | 0.408 |  | 1.41 | 0.13 | 14.85 | 0.773 |

Supplemental Table 7: Sexual behaviours and STIs associated with socialising with MSM in the last month using dating-specific apps, among MSM/TG who had socialised online in the last month

|  |  | **Socialised using Dating apps** | |  | **Crude** | | | |  | **Adjusted for place of birth, employment and education** | | | |
| --- | --- | --- | --- | --- | --- | --- | --- | --- | --- | --- | --- | --- | --- |
| **Nairobi, n=461 MSM/TG who have socialised online in the previous month** | | **n** | **RDS %** |  | **OR** | **95% CI** | | **p value** |  | **aOR** | **95% CI** | | **p value** |
| Overall socialising online using dating-specific apps in the previous month |  | 69/461 | 11.8 |  |  |  |  |  |  |  |  |  |  |
|  |  |  |  |  |  |  |  |  |  |  |  |  |  |
| Sex with a man in the past 3 months |  | 65/417 | 11.9 |  | 1.10 | 0.34 | 3.54 | 0.877 |  | 1.36 | 0.38 | 4.86 | 0.63 |
| Sex with a woman in the past 3 months |  | 11/116 | 5.9 |  | 0.39 | 0.17 | 0.91 | 0.030 |  | 0.45 | 0.19 | 1.06 | 0.07 |
| Condomless anal intercourse (3 months) |  | 32/214 | 10.6 |  | 0.81 | 0.43 | 1.54 | 0.522 |  | 0.82 | 0.41 | 1.64 | 0.58 |
| Receptive anal sex (3 months) |  | 45/259 | 12.1 |  | 1.07 | 0.56 | 2.02 | 0.844 |  | 1.35 | 0.66 | 2.76 | 0.40 |
| Number of sexual partners (3 months) | 0 | 4/44 | 11.0 |  |  |  |  | 0.192 |  | 1.00 |  |  | 0.19 |
|  | 1 | 7/104 | 5.4 |  | 0.47 | 0.11 | 1.99 |  |  | 0.62 | 0.13 | 2.92 |  |
|  | 2 | 20/121 | 13.0 |  | 1.21 | 0.35 | 4.22 |  |  | 1.62 | 0.41 | 6.39 |  |
|  | 3-5 | 19/131 | 14.8 |  | 1.41 | 0.40 | 4.99 |  |  | 1.56 | 0.41 | 5.96 |  |
|  | 6+ | 19/60 | 19.8 |  | 2.00 | 0.51 | 7.87 |  |  | 2.69 | 0.63 | 11.53 |  |
| Sold sex to a man (12 months) |  | 50/240 | 16.9 |  | 2.57 | 1.31 | 5.02 | 0.006 |  | 2.65 | 1.33 | 5.29 | 0.01 |
| Bought sex from a man (12 months) |  | 29/145 | 16.4 |  | 1.80 | 0.95 | 3.44 | 0.074 |  | 2.05 | 1.01 | 4.13 | 0.05 |
| STI symptoms (12 months) |  | 30/175 | 13.3 |  | 1.39 | 0.73 | 2.66 | 0.316 |  | 1.32 | 0.63 | 2.77 | 0.46 |
| CT/NG (rectal or urethral) |  | 15/123 | 10.6 |  | 0.86 | 0.40 | 1.81 | 0.684 |  | 0.93 | 0.41 | 2.09 | 0.86 |
| Syphilis (active) |  | 1/5 | 0.0 |  |  |  |  |  |  | 2.17 | 0.19 | 24.25 | 0.53 |
|  |  |  |  |  |  |  |  |  |  |  |  |  |  |
|  |  | **Socialised using Dating apps** | |  | **Crude** | | | |  | **Adjusted for age and religion** | | | |
| **Johannesburg, n=201 MSM/TG who have socialised online in the previous month** | | **n** | **RDS %** |  | **OR** | **95% CI** | | **p value** |  | **aOR** | **95% CI** | | **p value** |
| Overall socialising online using dating-specific apps in the previous month |  | 37/201 | 19.1 |  |  |  |  |  |  |  |  |  |  |
|  |  |  |  |  |  |  |  |  |  |  |  |  |  |
| Sex with a man in the past 3 months |  | 32/163 | 20.9 |  | 1.81 | 0.51 | 6.51 | 0.362 |  | 1.52 | 0.41 | 5.64 | 0.537 |
| Sex with a woman in the past 3 months |  | 6/47 | 6.3 |  | 0.21 | 0.08 | 0.60 | 0.004 |  | 0.21 | 0.07 | 0.62 | 0.005 |
| Condomless anal intercourse (3 months) |  | 16/89 | 21.2 |  | 1.28 | 0.53 | 3.10 | 0.588 |  | 1.14 | 0.46 | 2.85 | 0.773 |
| Receptive anal sex (3 months) |  | 21/105 | 23.7 |  | 1.76 | 0.72 | 4.32 | 0.218 |  | 1.57 | 0.62 | 3.95 | 0.339 |
| Number of sexual partners (3 months) | 0 | 5/38 | 12.7 |  |  |  |  | 0.503 |  | 1.00 |  |  | 0.531 |
|  | 1 | 9/61 | 16.9 |  | 1.40 | 0.33 | 5.98 |  |  | 1.08 | 0.26 | 4.51 |  |
|  | 2 | 9/50 | 17.9 |  | 1.50 | 0.35 | 6.54 |  |  | 1.27 | 0.28 | 5.64 |  |
|  | 3-5 | 8/36 | 32.7 |  | 3.34 | 0.72 | 15.43 |  |  | 3.01 | 0.63 | 14.34 |  |
|  | 6+ | 6/16 | 27.3 |  | 2.59 | 0.47 | 14.14 |  |  | 1.79 | 0.30 | 10.71 |  |
| Sold sex to a man (12 months) |  | 8/47 | 13.4 |  | 0.57 | 0.21 | 1.55 | 0.269 |  | 0.56 | 0.18 | 1.69 | 0.302 |
| Bought sex from a man (12 months) |  | 3/17 | 16.4 |  | 0.82 | 0.19 | 3.52 | 0.788 |  | 0.84 | 0.18 | 3.84 | 0.819 |
| STI symptoms (12 months) |  | 18/61 | 34.9 |  | 3.70 | 1.49 | 9.15 | 0.005 |  | 3.34 | 1.33 | 8.42 | 0.011 |
| Syphilis (active) |  | 4/23 | 27.4 |  | 1.74 | 0.50 | 6.04 | 0.383 |  | 1.27 | 0.35 | 4.62 | 0.717 |
| CT (urethral only) |  | 3/8 | 34.8 |  | 3.65 | 0.56 | 23.85 | 0.179 |  | 5.07 | 0.72 | 35.78 | 0.105 |
| NG (urethral only) |  | 2/3 | 44.7 |  | 2.30 | 0.19 | 27.25 | 0.510 |  | 12.78 | 0.68 | 238.92 | 0.090 |
